# The Type I IFN-Induced miRNA, miR-21

**DOI:** 10.3390/ph8040836

**Published:** 2015-11-25

**Authors:** Chuan He Yang, Kui Li, Susan R. Pfeffer, Lawrence M. Pfeffer

**Affiliations:** 1 Department of Pathology and Laboratory Medicine, University of Tennessee Health Science Center, 19 S. Manassas St., Memphis, TN 38163, USA; E-Mails: cyang@uthsc.edu (C.H.Y.); spfeffer@uthsc.edu (S.R.P.); 2 Center for Cancer Research, University of Tennessee Health Science Center, 19 S. Manassas St., Memphis, TN 38163, USA; 3 Department of Microbiology, Immunology and Biochemistry, University of Tennessee Health Science Center, 858 Madison Avenue, Memphis, TN 38163, USA; E-Mail: kli1@uthsc.edu

**Keywords:** IFN, miRNAs, cancer, antiviral

## Abstract

The interferon (IFN) family of cytokines not only has antiviral properties at various steps in the viral replication cycle, but also anticancer activity through multiple pathways that include inhibiting cell proliferation, regulating cellular responses to inducers of apoptosis and modulating angiogenesis and the immune system. IFNs are known to induce their biological activity through the induction of protein encoding IFN-stimulated genes. However, recent studies have established that IFNs also induce the expression of microRNAs (miRNAs), which are small endogenous non-coding RNAs that suppress gene expression at the post-transcriptional level. MiRNAs play critical roles in tumorigenesis and have been implicated to act as either oncogenes or tumor suppressors in various human cancers. Therefore, IFN-induced miRNAs play an important role, not only in the host response to innate immune response to cancer, but also in the tumorigenic process itself. Furthermore, IFN-induced miRNAs may participate in and/or orchestrate antiviral defense in certain viral infections. In this review, we describe our recent studies on the induction of miR-21 by type I IFN, the role of the STAT3 and NFκB signaling pathways in IFN-induced miR-21 expression, the role of miR-21 in different cancers and the role of miR-21 in regulating the antiviral response.

## 1. Introduction

The interferons (IFNs) are a family of endogenously-expressed glycoproteins initially discovered in 1957 based on their antiviral properties [1]. Although the IFN family is a central element in the innate immune response to infectious agents, it also has been found to have clinical efficacy in various nonviral human diseases. In fact, IFNs are currently among the most commonly-studied agents employed in the biological therapy of cancer. The anticancer action of IFNs involves the inhibition of cell proliferation by promoting cell cycle arrest [2], regulation of cellular responses to inducers of apoptosis [3], as well as modulation of angiogenesis and the immune system [4,5]. In addition, accumulating evidence suggests that IFN also plays an important role in the host response to cancer. For example, defects in the IFN system can lead to increased susceptibility to cancer through mechanisms that are incompletely understood [6].

Type I IFNs (IFNα and IFNβ) bind to a multisubunit cell surface receptor displayed on nearly all nucleated cells and elicit their biological effects by altering the pattern of target cell gene expression through the activation of the non-receptor protein tyrosine kinases, JAK1 and TYK2. These kinases induce the tyrosine phosphorylation of STAT proteins, which then travel into the nucleus and recognize promoter elements in IFN-stimulated genes (ISGs) to directly activate their transcription [7,8,9]. The focus of the IFN field has been on ISGs, which encode hundreds of proteins, and their subsequent role in IFN’s multiple biological activities [1,9,10,11,12,13,14,15]. However, a number of studies demonstrated that IFNs can also induce the expression of non-protein encoding RNAs, such as microRNAs (miRNAs) [16,17,18,19].

Mature miRNAs are an abundant family of small RNAs (~22 nucleotides) that are highly conserved throughout evolution [20]. Following the synthesis of long primary miRNA (pri-miRNA) transcripts by RNA polymerase II or III (hundred or thousand nucleotides long), nuclear processing by the enzyme Drosha produces a pre-miRNA transcript of ~70 nucleotides with a hairpin structure that is shuttled into the cytoplasm. The mature miRNA species is processed by the Dicer RNase in the cytoplasm into a 19- to 24-nucleotide product, which is then incorporated into the RNA-induced silencing complex (RISC). The RISC, using the ~7-nucleotide “seed sequence” of the miRNA, recognizes complementary sequences within the 3′ untranslated region (3′-UTR) of mRNA transcripts for translational suppression and/or degradation of the mRNA target. Over 1800 miRNA genes have so far been identified in the human genome that are believed to regulate an estimated 30% of all human genes [21]. Since each miRNA can simultaneously regulate the expression of tens to hundreds of genes, miRNAs function as “master-switches” to fine-tune gene expression post-transcriptionally and regulate multiple cellular pathways, such as embryonic development, immune response, inflammation and oncogenesis, as well as cellular growth and proliferation.

Since the IFN system is an integral part of the innate immune response, it is not surprising that IFN-regulated miRNAs have been implicated in this critical host defense pathway. IFNβ induces a number of miRNAs, which display seed sequences present in the hepatitis C virus (HCV) genome [16]. For example, miR-196 and miR-448 are induced by IFN in Huh7 hepatoma cells, and synthetic mimetics of these miRNAs attenuate HCV replication *in vitro* [16]. Several of these IFN-induced miRNAs (miR-1, miR-30, miR-128, miR-196, miR-296) are expressed in peripheral blood mononuclear cells (PBMCs) from healthy individuals and from chronic HCV-infected patients, and their expression is upregulated by IFN treatment to varying degrees [22]. These results indicate that IFN-induced miRNAs may participate in antiviral defense in certain viral infections. It is important to note, however, that more recent studies have questioned a physiological role that miRNAs may play in fending off viruses in vertebrate hosts, particularly because the majority of virus/IFN-induced miRNAs are present in low abundance that typically falls below the threshold (~100 copies per cell) believed to impart any biological functions (reviewed in [23]). In this review, we describe our recent studies on the induction of miR-21 expression by type I IFN, the role of the STAT3 and NFκB signaling pathways in IFN-induced miR-21 expression, the role of miR-21 in different cancers and the role of miR-21 in regulating the antiviral response.

## 2. MiR-21 Expression and Biological Functions

### 2.1. IFN-Induced miR-21 Expression

By bioinformatic analysis of miRNA promoters, we identified a potential binding site for STAT1 and/or STAT3 in the miR-21 promoter. In addition, miR-21 expression is upregulated by IL-6 and Toll-receptor signaling, which activate STAT3 [24,25]. To examine whether IFN affected miR-21 expression, human skin fibroblasts and prostate cancer, glioma and melanoma cancer cell lines were treated with IFNα/β; total RNA was isolated, and the expression of mature miR-21 transcript was determined by quantitative real-time PCR (qPCR) [18]. Although basal miR-21 expression was relatively low in normal human skin fibroblasts and varied among the different cancer cell lines, IFN induced a three to five-fold increase in miR-21 expression in all cells tested with the exception of PC3 prostate cancer cells. The inability of IFN to induce miR-21 expression in PC3 prostate cancer suggests a potential role of STAT3 in the regulation on miR-21 expression, since these cells lack the STAT3 gene [26]. IFN induced miR-21 expression at IFN concentrations above 10 units/mL and resulted in a near maximal induction at 100 units/mL. Moreover, IFN induced miR-21 expression within 2 h after IFN addition with levels peaking between 6 and 24 h after IFN addition and remaining elevated at 48 h after IFN addition. These findings almost parallel the dose-dependence and time course of IFN induction for the ISG, ISG15, indicating that there might be similarities in the IFN signaling pathway that leads to the induction of ISGs and miR-21.

### 2.2. Signaling Pathways in IFN-Induced miR-21 Expression

STAT3 was originally identified as a transcription factor for acute phase response genes and is activated by a wide variety of cytokines [15,27,28]. Under normal physiological conditions, STAT proteins are transiently activated with activation lasting anywhere from a few minutes to several hours. The phosphorylation of tyrosine 705 within the transactivation domain of STAT3 is required for STAT3 dimerization, nuclear translocation and induction of gene transcription. High persistent activation of STAT3 is found in diverse human tumors [29,30] and actively participates in tumor formation and progression [30]. The family of NFκB transcription factors binds to the promoters of genes, which play important roles in immunity, inflammation, cell growth and cell survival [31,32,33,34]. In mammals, the NFκB family includes NFκB1 (p105 processed to p50), NFκB2 (p100 processed to p52), RelA (p65), RelB and cRel. While p50 and p52 lack a transcription activation domain and, as homodimer, function as repressors, RelA, cRel and RelB have a transcription activation domain and, thus, when complexed with p50 or p52, are capable of activating transcription.

Several lines of evidence indicate that both STAT3 and NFκB signaling pathways regulate miR-21 expression. For example, Toll-like receptor activation by LPS upregulates miR-21 expression in macrophages, fibroblasts and PBMCs [35]. Moreover, IL-6 promotes the survival of multiple myeloma cells through the induction of miR-21 expression [25]. Both of these stimuli are known to activate STAT3 and NFκB signaling. As already described, IFN rapidly induced miR-21 expression in a dose-dependent manner [18]. However, while IFN robustly induced miR-21 expression in DU145 prostate cancer cells, IFN did not induce miR-21 expression in PC3 prostate cancer cells that have a genetic deletion of the STAT3 locus. We found that IFN-induced miR-21 expression in DU145 cells was ablated by the expression of a dominant negative STAT3 construct in which the required STAT3 tyrosine-phosphorylation site was mutated (F705-STAT3) or the knockdown of STAT3 expression [18]. Moreover, expression of wild-type STAT3 in PC3 cells restored the induction of miR-21 by IFN, but the expression of the dominant negative STAT3 construct did not. These results demonstrated that STAT3 is an important regulator of IFN-induced miR-21 expression. In addition, since miR-196a, miR-296 and miR-351 were found to be IFN-induced miRNAs [16], we explored the role of STAT3 in their IFN-induced expression. We found that while expression of F705-STAT3 also blocked the IFN induction of miR-351 and miR-296, it had no effect on IFN-induced miR-196a expression. Therefore, these results indicate that the expression of a subset of IFN-inducible miRNAs (miR-21, miR-351 and miR-296) is highly dependent on STAT3 activation.

Chromatin immunoprecipitation (ChIP) analysis of the miR-21 promoter showed that IFN induced STAT3 binding to the most distal STAT3 binding site [19], which was the same site in the miR-21 promoter that was previously found to regulate IL-6-induced STAT3 binding [25]. Taken together, these results demonstrate that the IFN induction of miR-21 is STAT3 dependent. Several potential NFκB binding sites are also present within the miR-21 promoter. Using mouse embryo fibroblasts deficient in the p65 NFκB subunit, we found that IFN induction of miR-21 was NFκB dependent [18]. By ChIP analysis, IFN was found to induce p65 NFκB binding to the miR-21 promoter in DU145 prostate cancer cells that was directly adjacent to the IFN-induced STAT3 binding site. Most interestingly, IFN-induced NFκB binding to the miR-21 promoter, as well as p65 nuclear translocation were ablated by STAT3 knockdown in DU145 cells. These results suggest that IFN induces the binding to the miR-21 promoter of a complex that contains both STAT3 and the p65 NFκB subunit. These findings are of particular interest, since we previously reported crosstalk between IFN-induced STAT3 and NFκB signaling pathways [14,36]. The crosstalk between the STAT3 and NFκB signaling pathways is associated with cancer and inflammation [37,38]. Moreover, enhanced miR-21 expression has been observed in various inflammatory conditions in which STAT3 and NFκB may also play critical roles [39,40,41,42].

### 2.3. The Role of miR-21 in Cancer

miR-21 is the most frequently overexpressed miRNA in human tumors and in cancer cell lines, and high miR-21 expression is observed in glioblastoma, head and neck cancer, ovarian cancer, B-cell lymphoma, hepatocellular carcinoma, cervical cancer and lung cancer [43,44]. miR-21 is associated with high proliferation, low apoptosis, high invasion and metastatic potential of cancer cells and promotes oncogenesis; hence, it has been called an oncomiR [24,25,45,46,47,48,49]. For example, in a study profiling 540 clinical samples from cancer patients, miR-21 was found to be the only consistently upregulated miRNA [46]. Induction of miR-21 expression in an inducible transgenic knock-in mouse model was found to result in spontaneous development of B-cell leukemia/lymphoma [50]. Moreover, miR-21 expression modulates tumor number, incidence and size in a K-ras-dependent mouse lung cancer model [43].

Based on previous studies indicating that miR-21 may regulate apoptosis, we examined the role of miR-21 in IFN-induced apoptosis. We previously showed that IFN induces apoptosis in some cancer cell lines, but this action appears to be counterbalanced by the induction of a potent cell survival pathway, which is NFκB dependent [51,52]. We next examined IFN-induced apoptosis in DU145 and PC3 cells and found that IFN induced marked apoptosis in PC3 cells, but induced little apoptosis in DU145 cells [18]. Since we found that IFN-induced miR-21 expression in DU145, but not in PC3 cells, we hypothesized that miR-21 might regulate IFN-induced apoptosis. To directly test this hypothesis, PC3 cells were transduced with miR-21, and stable cell lines were isolated with miR-21 levels comparable to DU145 cells. We then compared PC3 cells and those with restored miR-21 expression and found that miR-21 expression completely blocked IFN-induced apoptosis as measured by a cell death ELISA assay and by TUNEL staining. These results suggest that miR-21 expression suppresses IFN-induced apoptosis. Using a complementary approach to knockdown miR-21 expression in DU145 cells by ~85%, we found that IFN induced apoptosis in anti-miR-21 expressing DU145 cells. These results taken together suggest that miR-21 plays an important role in suppressing IFN-induced apoptosis.

To examine the role of miR-21 in a relevant *in vivo* model, we employed B16 melanoma cells, which are a well-studied model for the ability of melanoma to metastasize to the lung and form large metastatic lesions [53,54,55]. In brief, B16 cells were transduced with luciferase for live animal imaging and directly introduced into the bloodstream of syngeneic C57BL6 mice by tail-vein injection. Consistent with earlier studies, a strong luciferase signal was detected in the lungs of all mice injected with B16 cells. Most interesting, we found that, while B16 melanoma cells exclusively formed macrometatases in the lungs of tail-vein-injected mice, miR-21 KD (knockdown) B16 cells only formed smaller lung tumors, and tumors formed mostly in other tissues. Furthermore, the survival was markedly prolonged in mice injected with miR-21 KD cells [19].

A number of miR-21 target genes that play important roles in the oncogenic process have been previously identified, which include PTEN, PDCD4 and BTG2 [56]. We recently found that miR-21 regulates tumorigenesis in melanoma, glioblastoma and prostate cancer models and regulates the expression of these miR-21 target genes in a cell-type-specific manner [18,19]. We identified and validated that IGFBP3 and FBX011 are also miR-21 target genes, and thus, their expression is downregulated by miR-21. FBX011 (F-box only protein 11) is a component of the SCF ubiquitin ligase complex, targets BCL6 for degradation and is inactivated in diffuse large B-cell lymphomas [57]. The insulin-like growth factor signaling pathway involves two ligands (IGF-I and IGF-II), three surface receptors (IGF1R, IGF2R and IGF3R) and six IGF-binding proteins (IGFBPs) and plays an important role in growth, development and the maintenance of homeostasis in normal cells. Accumulating evidence suggests that disruption of the IGF system has major implications for growth retardation, atherosclerosis, insulin resistance and cancer [58]. The involvement of IGFBPs in cancer varies depending on the type of malignancy. For example, serum levels of IGFBP3 have been reported to be associated with disease progression in melanoma patients [59].

### 2.4. The Role of miR-21 in Regulating IFN-Mediated Antiviral Action

**Figure 1:**
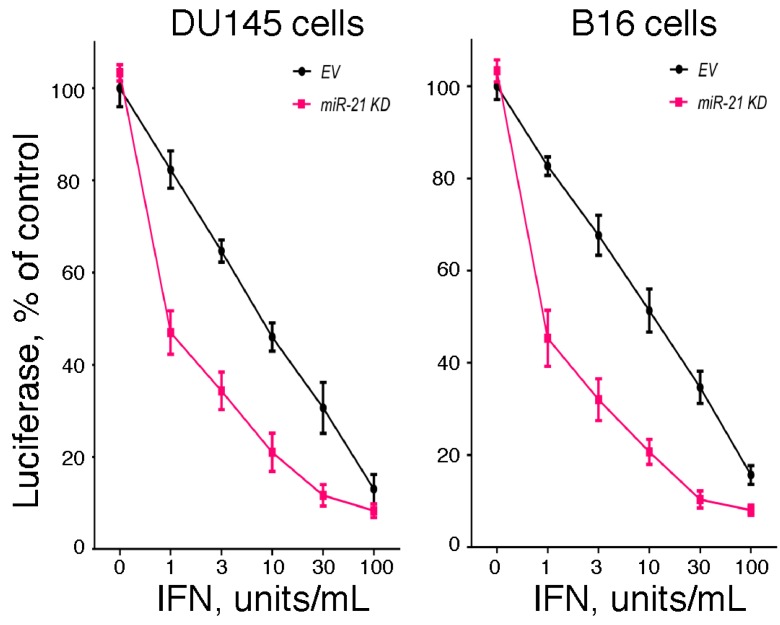
The role of miR-21 in the antiviral action of IFN. Empty vector (EV)- and antagomiR-21 transduced (miR-21 KD) human DU145 prostate cancer cells or murine B16 melanoma cells were added to 48-well plates (1 × 10^4^ cells/well). After incubation overnight, triplicate wells were treated with 0, 1, 3, 10, 30 or 100 units/mL of human IFNα or murine IFNβ, respectively, for an additional 24 h. The cells were then infected with a recombinant vesicular stomatitis virus expressing firefly luciferase (rVSV-Luc) as the readout for viral replication (generously provided by Dr. Sean Whelan, Harvard Medical School) at a multiplicity of infection of one. At 6 h post-infection, cells were lysed by the addition of 65 μL of lysis buffer, and luciferase activity in the cell lysate (10 μL) was determined.

**Figure 2:**
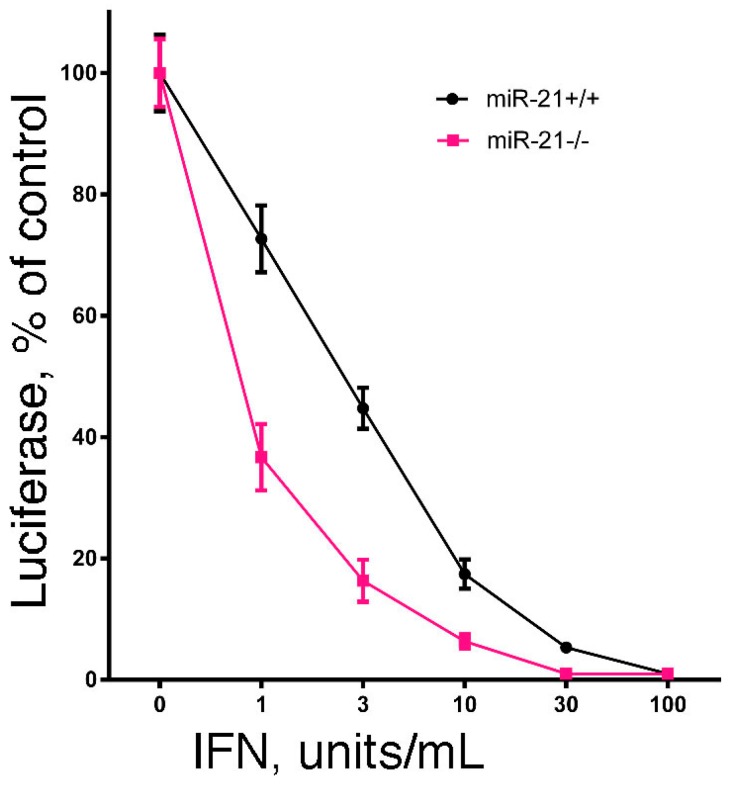
The effect of miR-21 deficiency on the sensitivity of mouse embryo fibroblasts to the antiviral action of IFN. Mouse embryo fibroblasts from miR-21 knockout mice or their wild-type littermates (generously provided by Dr. Mark Hatley, St. Jude Children’s Research Hospital, Memphis, TN, USA) were added to 48-well plates (1 × 10^4^ cells/well). After incubation overnight, triplicate wells were treated with 0, 1, 3, 10, 30 or 100 units/mL of murine IFN for an additional 24 h. The cells were then infected with rVSV-Luc at a multiplicity of infection of one. At 6 h post-infection, cells were lysed by the addition of 65 μL of lysis buffer, and luciferase activity in the cell lysate (10 μL) was determined.

Since the hallmark of IFN is its antiviral activity, we examined the effect of miR-21 expression on IFN-induced antiviral activity. In brief, we examined the IFN-induced antiviral action against vesicular stomatitis virus in DU145 and B16 cells after knockdown of miR-21 expression using a highly sensitive luciferase-based reporter assay [60]. As shown in Figure 1, knockdown of miR-21 in both cell lines markedly sensitized the cells to IFN-induced antiviral action. These results suggest that miR-21 dampens the sensitivity of cells to the antiviral activity of IFN. In addition, as a complementary approach, we examined the IFN-induced antiviral action in mouse embryo fibroblasts derived from miR-21 knockout mice [60]. As shown in Figure 2, and consistent with our findings in miR-21 knockdown cells, miR-21-defective mouse embryo fibroblasts were sensitized to the antiviral activity of IFN.

### 2.5. The Role of miR-21 in Regulating Immune Mechanisms Leading to IFN Expression

Several studies have shown that miR-21 can shape the host innate immune response. For example, miR-21 expression is rapidly upregulated in hepatoma Huh7 cells following HCV infection [61]. Interestingly, miR-21 was found to target two important components, MyD88 and IRAK1, in the Toll-like receptor signaling pathway that leads to type I IFN production. By downregulating MyD88 and IRAK1 expression in Huh7 cells through a miR-21-mediated pathway, HCV represses type I IFN production and promotes viral infection. In addition, miR-21 plays a central role in setting the balance of Th1 and Th2 cytokine expression. For example, targeted ablation of miR-21 in mice enhanced the production of the Th1 cytokine IFNγ, as well as the delayed-type hypersensitivity cutaneous response [62]. Moreover, miR-21 knockdown in primary human lymphocytes resulted in enhanced IFNγ production and strengthened T-cell activation, indicating that miR-21 is a negative regulator of T-cell activation [63]. Future studies will elucidate whether miR-21 is a negative regulator of type I IFN (IFNα/β) production in activated T-cells.

## 3. Conclusions

IFNs are well known to induce their diverse biological actions through the induction of hundreds of ISGs. However, recent studies show that IFNs also induce the expression of miRNAs that may fine-tune the expression of multiple genes at the post-transcriptional level. In this review, we describe that: (1) miR-21 expression is rapidly induced upon IFN treatment; (2) the time course and dose dependence of miR-21 expression is highly similar to that of ISGs; (3) IFN-induced miR-21 expression is dependent on both the STAT3 and NFκB signaling pathways; (4) STAT3 and the p65 subunit of NFκB bind to the miR-21 promoter and regulate its expression; (5) IGFBP3 and FBX011 are direct miR-21 target genes, and thus, their expression is downregulated by miR-21; (6), miR-21 promotes the oncogenesis of various cancers; and (7) miR-21 suppresses the IFN-mediated antiviral response and regulates immune mechanisms, leading to IFN expression. Future studies should elucidate the role of other IFN-induced miRNAs, their target genes and the role of these miRNAs and their target genes in diverse biological actions of IFNs.
